# Mycetoma laboratory diagnosis: Review article

**DOI:** 10.1371/journal.pntd.0005638

**Published:** 2017-08-24

**Authors:** Amel Altayeb Ahmed, Wendy van de Sande, Ahmed Hassan Fahal

**Affiliations:** 1 The Mycetoma Research Centre, University of Khartoum, Khartoum, Sudan; 2 Department of Medical Microbiology & Infectious Diseases, Erasmus MC, University of Rotterdam, Rotterdam, the Netherlands; University of Queensland, AUSTRALIA

## Abstract

Mycetoma is a unique neglected tropical disease caused by a substantial number of microorganisms of fungal or bacterial origins. Identification of the causative organism and the disease extension are the first steps in the management of the affected patients and predicting disease treatment outcome and prognosis.

Different laboratory-based diagnostic tools and techniques were developed over the years to determine and identify the causative agents. These include direct microscopy and cytological, histopathological, and immunohistochemical techniques in addition to the classical grain culture. More recently, various molecular-based techniques have joined the mycetoma diagnostic armamentarium. The available mycetoma diagnostic techniques are of various specificity and sensitivity rates. Most are invasive, time consuming, and operator dependent, and a combination of them is required to reach a diagnosis. In addition, they need a well-equipped laboratory and are therefore not field friendly.

This review aims to provide an update on the laboratory investigations used in the diagnosis of mycetoma. It further aims to assist practising health professionals dealing with mycetoma by outlining the guidelines developed by the Mycetoma Research Centre, University of Khartoum, WHO collaborating centre on mycetoma following a cumulative experience of managing more than 7,700 mycetoma patients.

## Introduction

Mycetoma is a devastating chronic subcutaneous granulomatous inflammatory disease caused by several true fungi and bacteria, and hence it is classified as eumycetoma and actinomycetoma, respectively [1,2]. The disease is characterised by numerous deformations and disabilities, high morbidity, and in its late stage it is potentially fatal. Mycetoma is endemic in the so-called “mycetoma belt” that includes various countries across the world, but it is reported extensively from Sudan, Mexico, and India [3,4].

**Fig 1:**
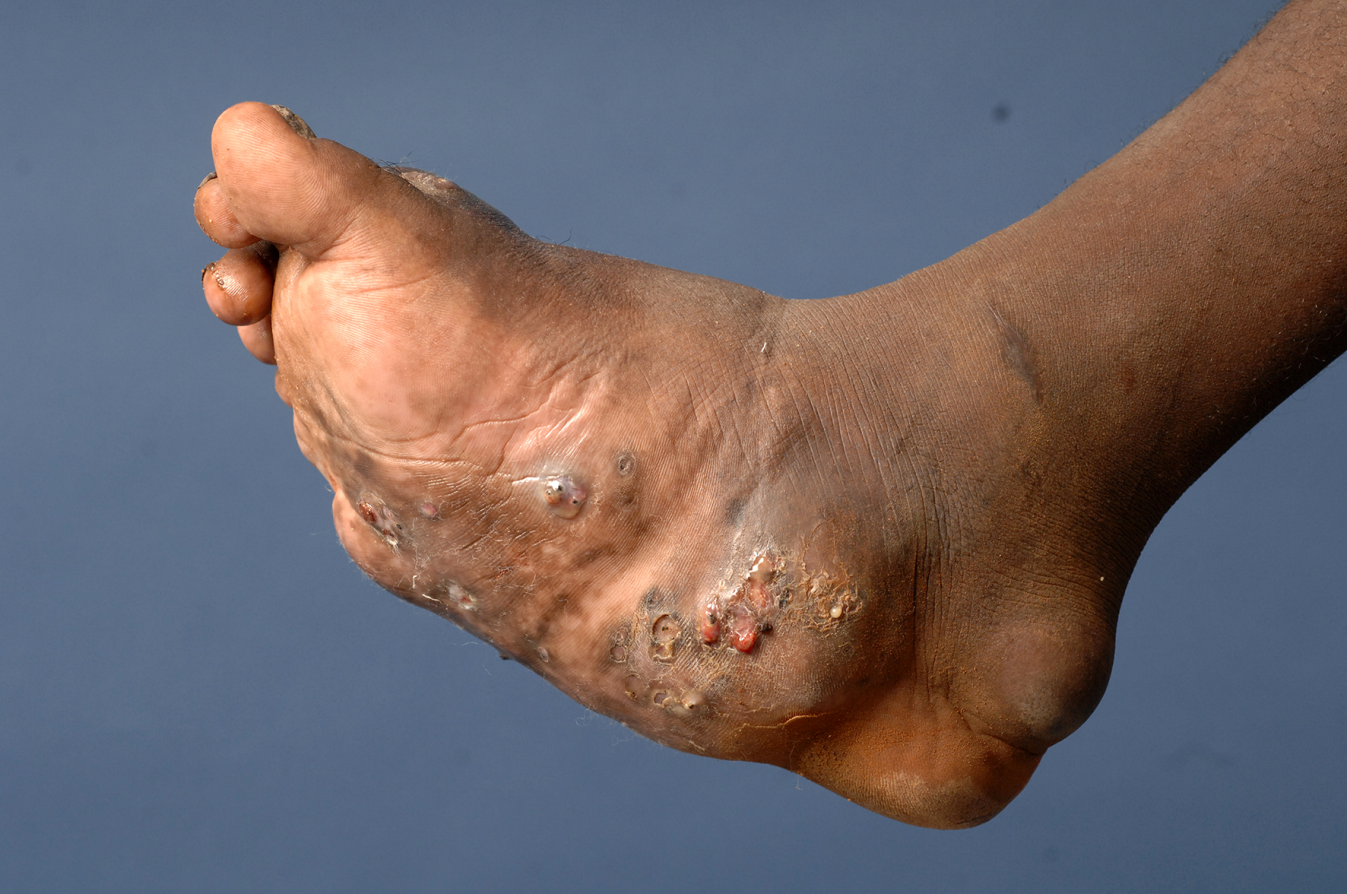
Photography showing the mycetoma triad of mass, multiple discharging sinuses, and black grains.

The triad of a painless subcutaneous mass, multiple sinuses, and discharge that contains grains of different colours, sizes, and consistency is characteristic of mycetoma [2]. Late presentation of the majority of patients is the norm, and the explanation is multifactorial, with reasons including the painless nature of the disease, patients’ low socioeconomic status and health education, and scarcity of health facilities in endemic regions (Fig 1) [3,5,6,7].

Mycetoma is classified as eumycetoma and actinomycetoma, and they are caused by a considerable number of microorganisms of both fungal and bacterial origin, respectively (Table 1).

**Table 1:** The common different mycetoma causative organisms.

*Grain color*	*Causative organisms*	*Diagnosis*
**Eumycetoma**		
Black grains	*Madurella* spp.	Histopathological examination
	*Leptosphaeria* spp.	PCR
	*Curvularia* spp.	
	*Exophiala* spp.	
	*Phaeoacremonium* spp.	
	*Phialophora verrucosa*	
	*Pyrenochaeta mackinnonii*	
	*P*. *romeroi*	
Pale, white, yellow grains	*Pseudallescheria boydii (Scedosporium apiospermum)*	
	*Acremonium* spp.	
	*Aspergillus s*pp.	
**Actinomycetoma**		
Pale, white, yellow grains	*Actinomadura madurae*, *Nocardia* spp.	Histopathological examination
Yellow to brown grains	*Streptomyces* spp.	PCR
Red to pink grains	*A*. *pelletierii*	

Hence, proper treatment of mycetoma requires adequate and accurate diagnosis of the causative organisms. Currently, a series of investigations are available to establish the diagnosis of mycetoma [8]. Most of these investigations are invasive, time consuming, and require good personal experience. In most instances, a combination of these investigations is required in a well-equipped laboratory to reach a diagnosis [8]. The aim of this article is to discuss the pros and cons of the available laboratory-based diagnostic investigations in order to assist treating clinicians with requesting appropriate investigations.

### Mycetoma grains

**Fig 2:**
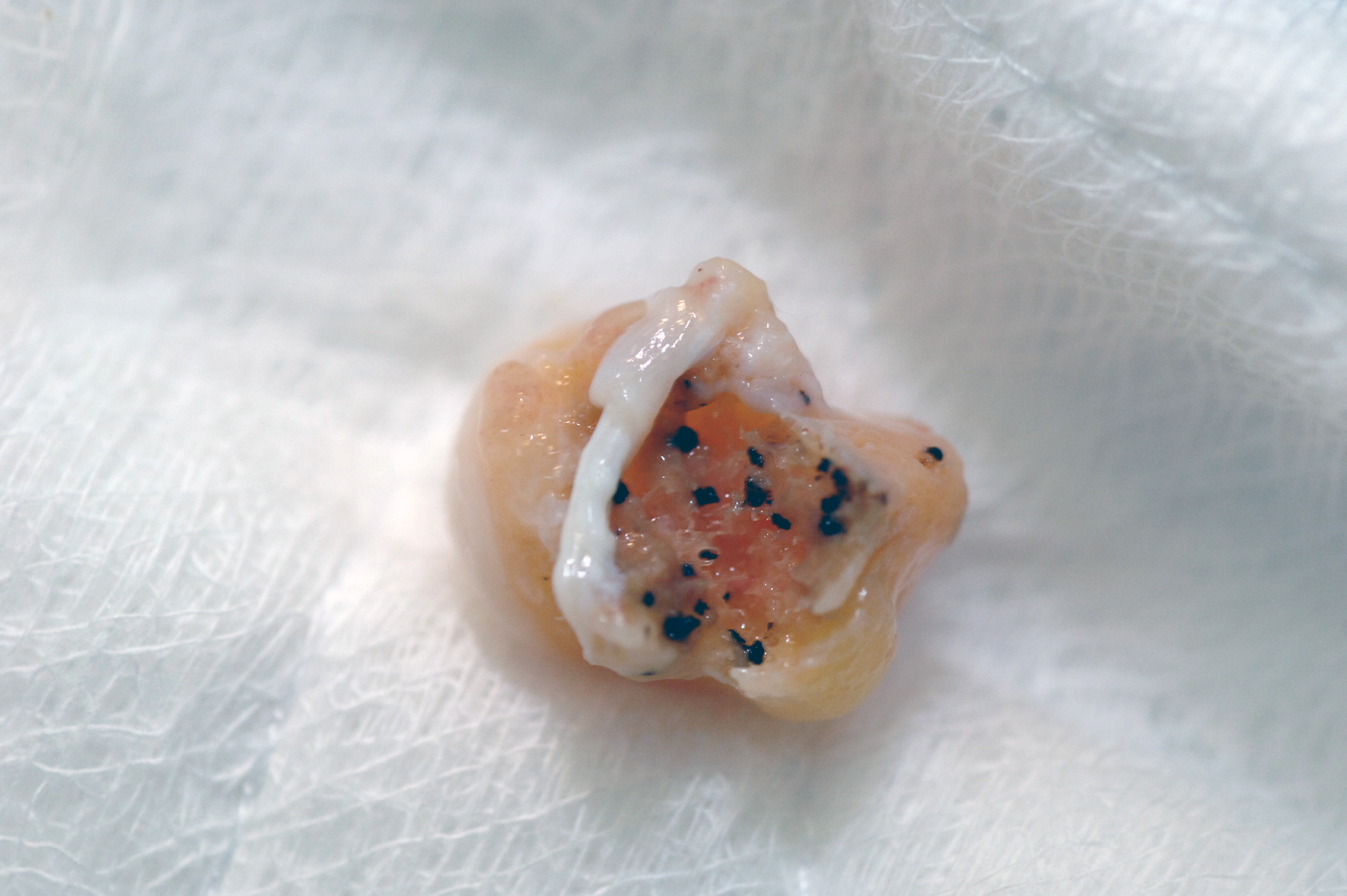
Photography of surgical biopsy showing a well-encapsulated eumycetoma lesion with numerous black grains.

Grains are crucial to establish the diagnosis of the causative organism. Although the grains’ morphological characteristics may provide a rapid provisional identification of the aetiological agent, in some instances they may be deceiving [1,4,5,8]. Grains have different morphological features; their size varies from microscopic to 1–2 mm in diameter. *Madurella mycetomatis* and *A*. *madurae* have large grains, whereas *Nocardia brasiliensis*, *N*. *cavae*, and *N*. *asteroids* grains are small in size [8–11]. Their colour is variable, ranging from black, yellow, white, or red to pale. Most of eumycetoma causative organisms produce black or pale grains and rarely yellow, and actinomycetoma commonly is caused by organisms that produce yellow, white, or red grains [10]. The consistency of most grains is soft, but *Streptomyces somaliensis* and *M*. *mycetomatis* are quite hard (Fig 2) [1].

Grains are commonly obtained by deep surgical biopsies under aseptic conditions to avoid contamination. Grains obtained from open sinuses are commonly not viable and often contaminated [10].

The surgical biopsies must be handled immediately by the laboratory technologist in the surgical theatre. The biopsy should be divided into 2 parts; 1 part for the grains culture and the other for histopathological examinations. The former is placed in normal saline, while the latter is placed in 10% formal saline.

### Grains direct microscopy

Direct microscopic examination of grains obtained from the sinuses’ serosanguineous discharge is the fastest means of making a presumptive diagnosis of the mycetoma causative organisms, but it lacks accuracy. The grains can be directly examined under light microscope using 10% potassium hydroxide (KOH), which digests the mucus and keratin, thus providing a clear background [9,10, 11,12]. Direct grain microscopy can rule out actinomycotic causative agents. However, it cannot discriminate between certain organisms such as *M*. *mycetomatis* and *Trematosphaeria griesia*. Therefore, this procedure is not specific enough to make a definitive diagnosis of mycetoma and should be supplemented by additional characteristic features identification. In addition to the 10% KOH, Parker ink can be used to examine the serosanguineous discharge containing grains microscopically.

**Fig 3:**
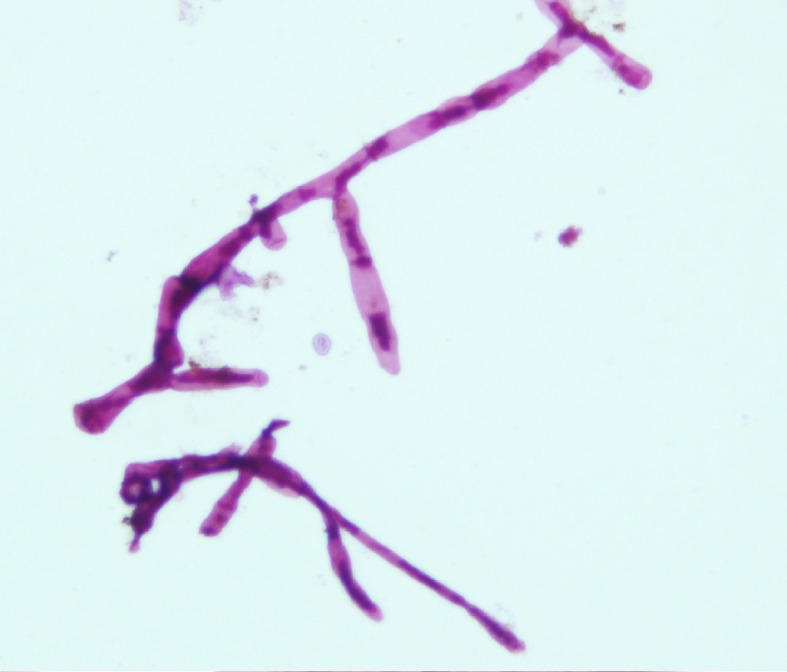
KOH wet mount direct microscopic examination of *M. mycetomatis* grains showing its hyphal structure.

The crushed grains are placed on a glass slide and covered with a coverslip. Two or 3 drops of the stain are applied to the slide edge and allowed to infiltrate under the slip. The preparation is then examined under light microscope for the presence of the hyphae and spores. They usually take up a dark blue colour in a light blue cellular background. Actinomycetes under the microscope usually show branching filaments, abundant aerial mycelium, and long chains of spores (Fig 3).

Fabric fibres can cause diagnostic confusion, as they can also take up the stain, but they are often out of the tissue plane, larger than the hyphae, irregular in diameter, and often have an irregular spiral configuration [9,10].

Several histochemical staining techniques are used for rapid identification of mycetoma causative agents from culture. Gram staining and the Ziehl–Neelsen (ZN) staining technique are the commonest in use. Actinomycetoma causative organisms are gram-positive, while eumycetoma causative organisms are gram-negative [9,10,12]. The actinomycetes consist of fine, branching filaments, about 1 micron thick, whereas the eumycetes grains are composed of septate hyphae 4–5 microns thick. The ZN staining technique is superior in discriminating between actinomycotic agents; *Nocardia* spp. are ZN positive, whereas *A*. *madurae* and *S*. *somaliensis* are ZN negative [9, 10,12].

### Grains culture

A sizeable number of grains are needed to culture the causative agents of mycetoma. They must be soaked and stored in saline for culture, washed several times with normal saline, and inoculated onto suitable culture media in sterilised conditions using either a safety cabinet or a flame-sterilised area. Modified Sabouraud agar supplemented with 0.5% yeast extract, blood agar, brain–heart infusion agar, and Löwenstein agar are the commonly recommended media.

Antibiotic free culture media is required for the isolation of actinomycetes, while the eumycetes culture must contain antibiotics. The commonly used antibiotics are penicillin G (20 U/ml), gentamicin sulphate (400 μg/ml), streptomycin (40 μg/ml), or chloramphenicol (50 μg/ml).

The mycetoma causative organisms can be identified by their textural description and morphological and biological activities in pure culture. The biological activity may include acid fastness, optimal temperature, proteolytic activity, utilisation of sugars, and nitrogenous compounds [12].

**Fig 4:**
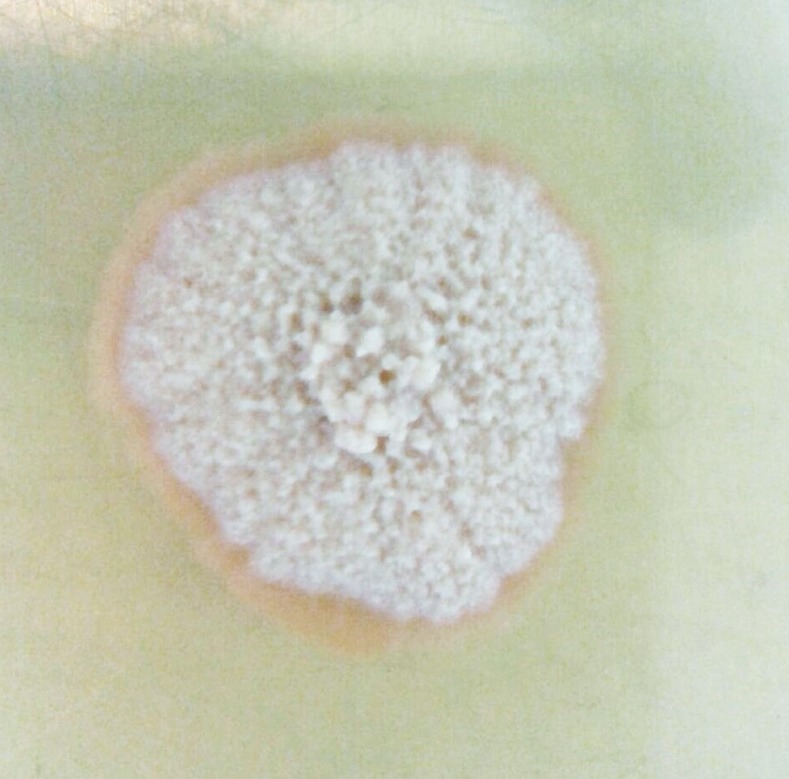
Photograph showing *M*. *mycetomatis* growth in Sabouraud agar media.

*Nocardia* typically produce substrate and aerial hyphae that look like rods and coccoids. *Streptomyces* form a yellowish substrate mycelium and lack aerial hyphae, while identification of *Madurella* is mainly based on the morphology of the fruiting bodies and morphology of the colonies (Fig 4) [9,10,12,13].

The phenotypic characteristics of the causative organisms are important for their identification. These include the production of β-glucuronidase; degradation of adenine, casein, and hypoxanthine; growth on adonitol; aesculin hydrolysis; glycerol; glycogen; D-raffinose; L-rhamnose; D-turanose; D-xylose; and L-aspartic acid. The latter is a sole carbon source and plays a role in the identification of pathogenic *Streptomyces* spp. *A*. *madurae* was found to be positive for α-glucosidase and negative for *N*-acetyl-β-glucosaminidase [9,10,12].

In view of the limited information available on phenotypic properties and assimilation patterns for the identification of eumycetoma agents, a new system, the API 20C AUX kit, was recently introduced and was able to identify *M*. *fahalii*, *M*. *pseudomycetomatis*, and *M*. *tropicana* [14].

Differentiation of the various dematiaceous fungi on the basis of morphology is sometimes difficult and time consuming, and culture usually takes about 3 weeks to give an accurate result. However, the culture technique is time consuming, and accidental contamination may give a false positive result. It also requires experience to identify the causative organisms [3,4,12,15].

### Mycetoma cytological identification

**Fig 5:**
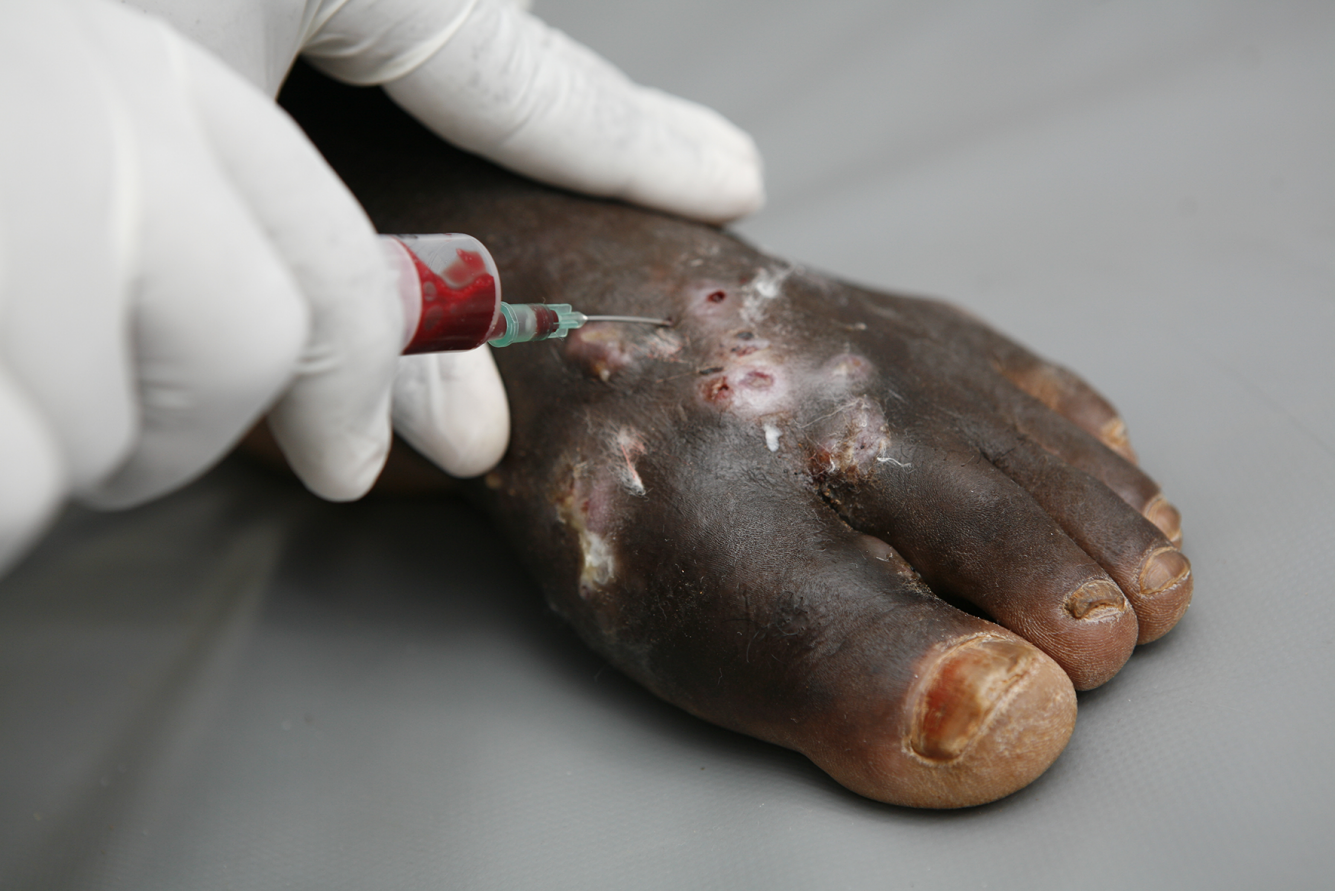
Photograph showing the fine-needle aspiration cytology collection technique.

Fine-needle aspiration cytology (FNAC) with cell blocks and imprint cytology techniques for mycetoma were described [16,17]. Fine-needle aspirations under aseptic conditions is required to identify the causative agent of mycetoma and the tissue reaction against it. In this technique, a needle attached to a syringe is inserted into the suspected mycetoma lesion and aspirated under negative pressure. It should be performed in at least 3 different directions (Fig 5). After fixation, the cell blocks are processed and stained as is done with the paraffin sections. The smears or the sections can be examined microscopically.

Mycetoma has characteristically distinct cytological features, characterised by the presence of suppurative granulomas surrounding the characteristic grains of the causative organism. The granuloma consists of neutrophilic infiltrate in close contact with and infiltrating the grains. It is surrounded by palisading histiocytes beyond which there is a mixed inflammatory infiltrate comprising lymphocytes, plasma cells, eosinophils, macrophages, and fibrosis. Multinucleated giant cells are frequently encountered in the granuloma.

**Fig 6:**
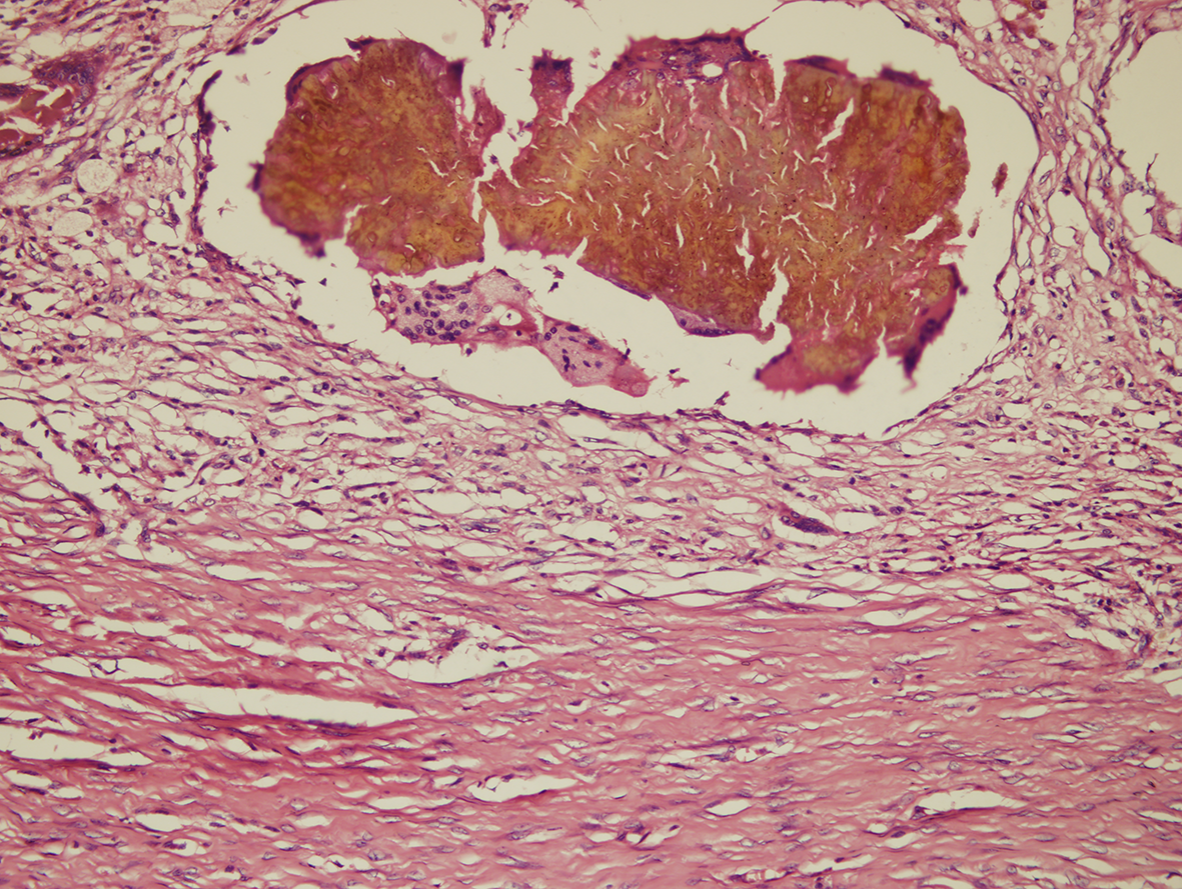
Photomicrograph showing *M. mycetomatis* and inflammatory infiltrate in a cytological smear. Haematoxylin and eosin x 40.

In smears stained with haematoxylin and eosin (H&E), the *M*. *mycetomatis* grains appeared rounded or oval in shape and black with a green tinge of colour or occasionally brownish. Two types of *M*. *mycetomatis* grains can be identified in cytological smears: the solid granular type, which is the commonest, and the vesicular type. The septate hyphae are not identified in the first type because they are embedded in a hard, brown cement matrix. The vesicular type consists of swollen fungal cells and are seen as vesicles (Fig 6).

Actinomycetes grains are homogeneously eosinophilic in H&E. In Geimsa-stained smears, the grain appears homogeneously blue in the centre, while in the periphery it consists of fine granules and radiating pink filaments. The *A*. *pelletierii* grain is more eosinophilic in H&E as compared to *S*. *somaliensis*, and it is semilunar in shape, as seen in histology.

The cytological smears can differentiate mycetoma from other subcutaneous lesions and can identify the mycetoma causative agents. The technique is simple, quick, economical, and can be used for sample collection in epidemiological surveys and culture. The presence of grains in the cytological smear is mandatory to reach a diagnosis. The technique is noted to be painful in some patients and may induce infection and cellulitis in the area [16,17].

EL Hag and colleagues in 1995 studied a group of 14 patients with different types of mycetoma lesions using the FNAC technique. The findings from the cytological smears were comparable to those observed in histological sections. They concluded that this technique is useful for the routine diagnosis of mycetoma in epidemiologic surveys and for material collection [16]. Yousif and colleagues in 2009 used FNAC and the cell block technique in the diagnosis of 230 patients with different types of mycetoma, and they reported sensitivity rates of 87.5% and 85.7% for eumycetoma and actinomycetoma identification, respectively [17].

In summary, simple and inexpensive cytological techniques such as FNAC and imprint smears that employ routine H&E, May–Grünwald–Giemsa, Papanicolaou, and periodic acid–Schiff (PAS) stains on cytological specimen usually lead to rapid diagnosis of mycetoma, particularly in remote, endemic regions.

### Histopathological and histochemical techniques

Surgical biopsies are commonly obtained by wide local excision or deep incisional biopsy, and currently a Tru-Cut needle biopsy is in use. Surgical biopsies taken under local anaesthesia are painful and usually yield inadequate specimens and have to be avoided [6].

The initial step is the fixation of biopsy samples in suitable fixatives such as 10% formal saline. The tissue processing then follows several steps: dehydration by different concentrations of ethanol, clearing by Xylene, impregnation and embedding by using paraffin wax (a 2- to 5-micron-thick section obtained by microtome), and finally staining with different staining techniques [12,18,19].

H&E is the stain for primary identification of the causative agent and the tissue reaction. Special stains usually follow for accurate identification of certain organisms and the cell components such as proteins, lipids, carbohydrates, and minerals that can be associated with the disease.

The special stains commonly in use are Grocott’s hexamine silver, PAS, Masson–Fontana stain, Perl’s Prussian blue, von Kossa’s stain, formalin-induced fluorescence, and Schmorl’s stain. Modified bleaching technique is also in use [12,18,19].

Actinomycete grains can be identified by Gram and ZN stains [12,18,19]. H&E, PAS, Grocott’s methenamine silver, and Gridley are the most useful stains for detecting hyphae and chlamydospores in eumycetes grains [12, 18,19].

Under microscopy, the fungal structures are broad, septate, and branching hyphae with large swollen cells at the edge. The hyphae may be hyaline or pigmented. Grain cement may or may not be seen, and if present, it may be compact or loose. These characteristics are useful for identification but may not be used for definitive diagnosis [18,19].

The *M*. *mycetomatis* grains are large, ranging from 0.5 to 3 mm; appear rounded, oval, or trilobed; and consist of intertwining hyphae embedded in interstitial brownish cement. The cement contains melanin, heavy metals, proteins, and lipids [18,19]. The grain can consist of filamentous or vesicular types. Brown septate and branched hyphae that may be slightly more swollen in the periphery are indicative of a filamentous grain, while unusually large cells that look like vesicles predominate in the vesicular type [18,19,20].

The host tissue reaction against the mycetoma causative organisms is distinctive, [21,22]. There are 3 types of tissue reactions.

### Type I reaction

**Fig 7:**
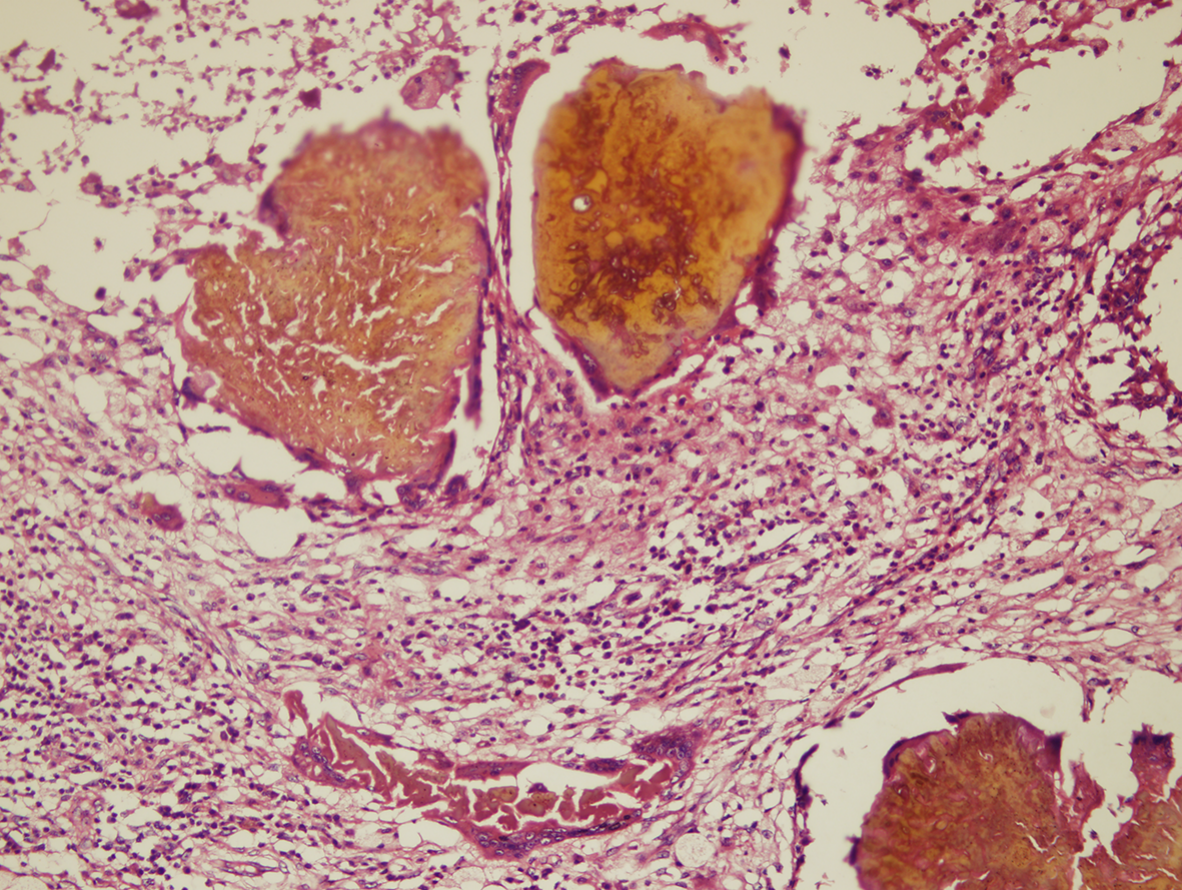
Photomicrograph showing a *M*. *mycetomatis* grain with a type I tissue reaction. Haematoxylin and eosin x 10.

In this reaction, the grains are surrounded by a layer of polymorphonuclear leukocytes, with neutrophils closely attached to the surface of the grain or invading the substance of the grain. This is surrounded by a layer of cells consisting of plasma cells, macrophages, lymphocytes, and a few neutrophils in a granulation tissue. Layers of fibrin usually surround the venules and the capillaries, and the outermost layer of the lesion contains fibrous tissue (Fig 7).

### Type II reaction

**Fig 8:**
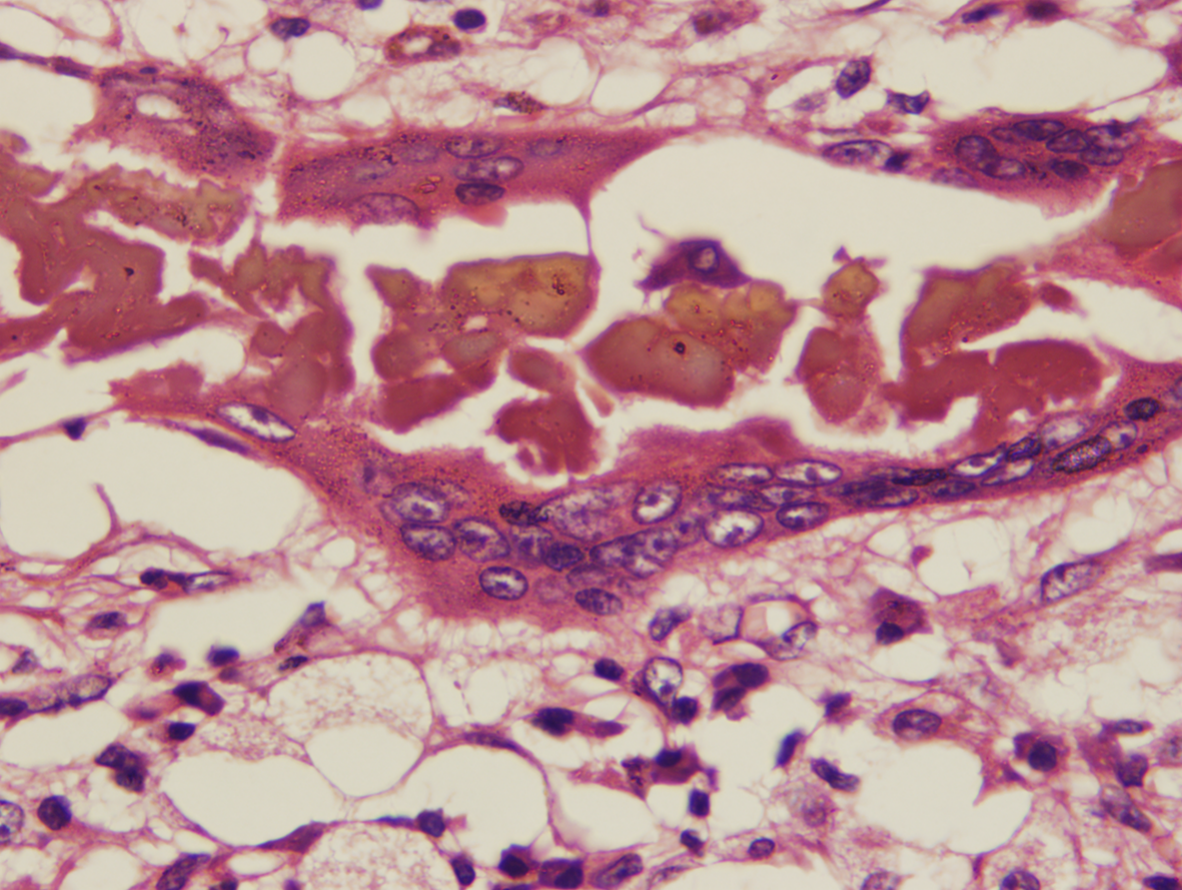
Photomicrograph showing *M*. *mycetomatis* and a type II tissue reaction. Haematoxylin and eosin x 10.

In this reaction, the macrophages and multinucleated giant cells replace most of the neutrophils. Fragments of the destroyed grains are commonly seen within the multinucleated giant cells (Fig 8).

### Type III reaction

**Fig 9:**
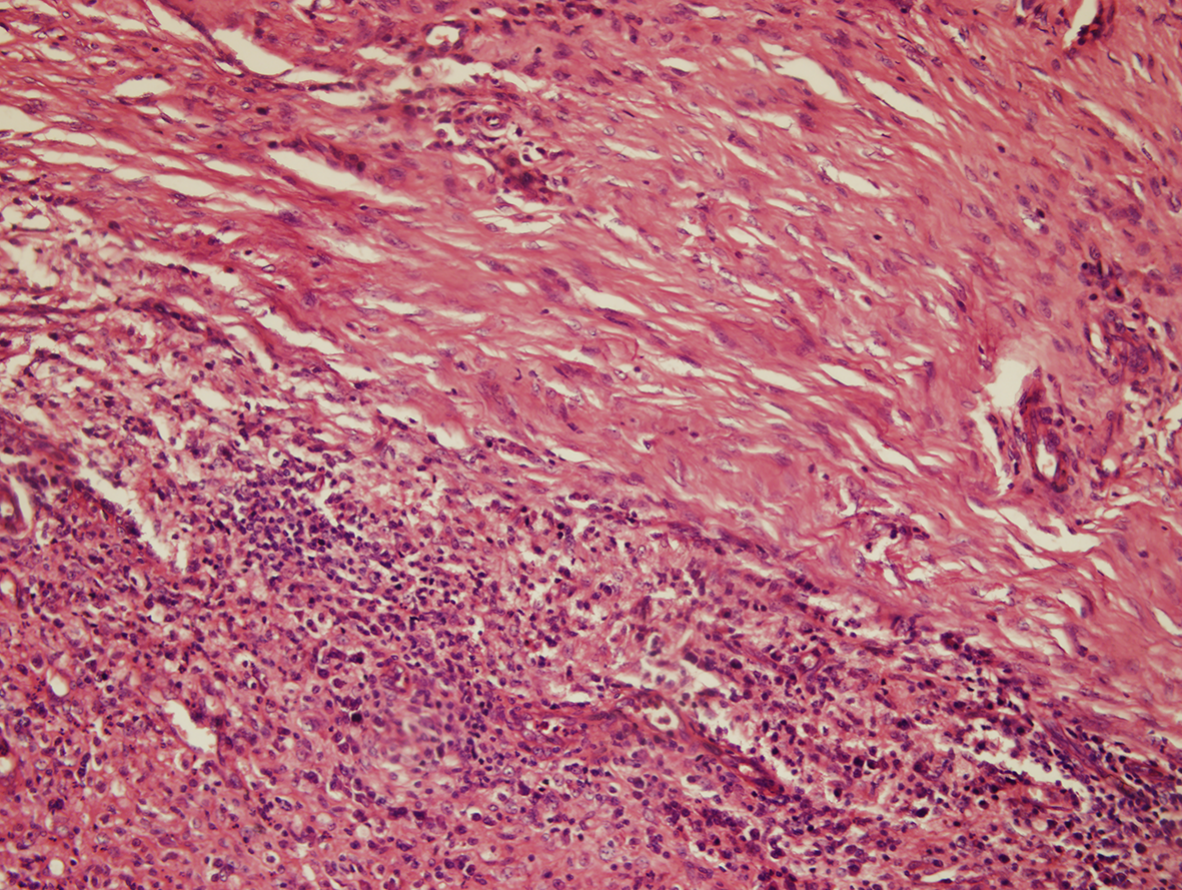
Photomicrograph showing a type III tissue reaction. Haematoxylin and eosin x 10.

This reaction is commonly characterised by well-organized epithelioid granulomas containing Langerhans giant cells, and usually no grains are seen (Fig 9).

**Fig 10:**
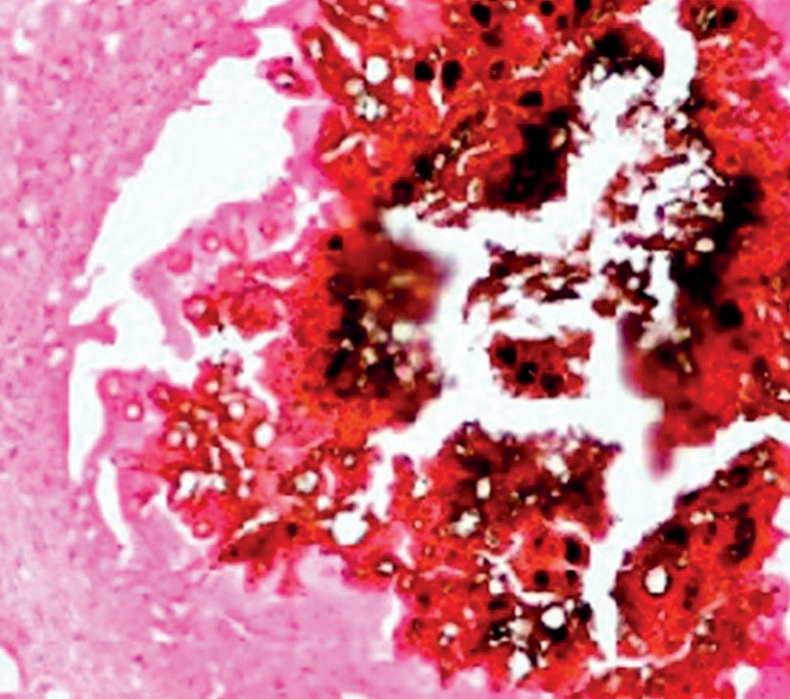
Photomicrograph showing *M*. *mycetomatis* hyphae and cement substances that are positive for calcium stained with von Kossa stain.

The technique is remarkable, as it does not require aseptic technique or need an inflexible time frame. However, accurate identification of certain organisms proves to be difficult, it lacks the culture precision, and it takes several days to deliver the results. A deep biopsy that contains grains is always needed to establish a diagnosis (Fig 10).

### Serodiagnosis

**Fig 11:**
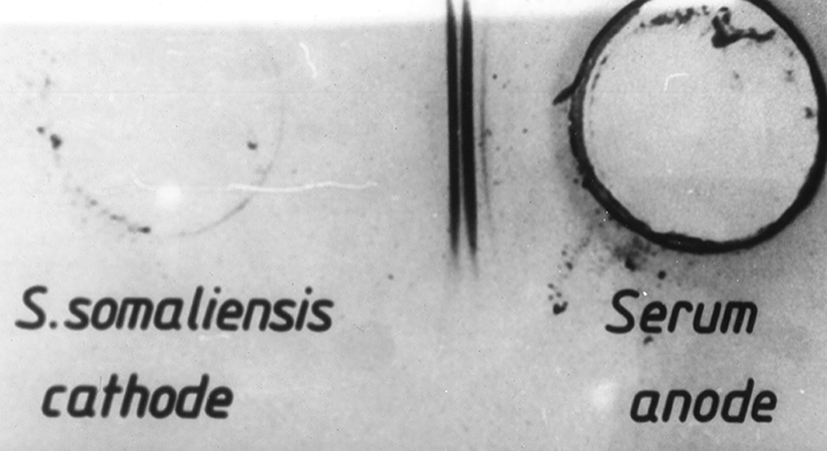
Photograph showing a counterimmunoelectrophoresis test with positive bands.

Over the years, different serological tests and assays were used for the diagnosis of mycetoma. These included immunoblots, indirect haemagglutination assays (IHAs), immunodiffusion (ID), counterimmunoelectrophoresis (CIE), and ELISA (Fig 11) [8,23–27].

Salinas-Carmona and his associates reported on the use of ELISA for the serological diagnosis of *N*. *brasiliensis*, the most common agent causing actinomycetoma in Mexico [23]. This study revealed a higher incidence of antibodies in patients with active disease without cross-reactions with *Mycobacterium leprae* and *M*. *tuberculosis*. This has been useful in cases in which identification of the aetiological agent in culture was not possible.

For eumycetoma agents, serological assays have been developed only for *M*. *mycetomatis* and *P*. *boydii*. For *P*. *boydii*, both an ID assay with crude antigens and an IHA were developed. The ID assay and CIE test for *M*. *mycetomatis* were most widely applied using crude cytoplasmatic antigens [24,25]. CIE was superior to ID since lower antibody titres were found. Unfortunately, since crude antigen preparations were used, cross-reactivity occurred and reproducibility was low [24,25]. The only serological assays performed with pure antigens of *M*. *mycetomatis* were an ELISA based on the recombinant-produced translationally controlled tumour protein (TCTP) and the Luminex assays based on TCTP, fructose-bisphosphate aldolase (FBA), and pyruvate kinase (PK) [27,28]. Although patients had higher levels of antibodies, the same levels were also detected in healthy controls, making the techniques unsuitable as diagnostic tools [27,28].

ELbadawi and associates in 2016 reported on the 75% sensitivity and 95% specificity of immunoblotting of *M*. *mycetomatis* cytoplasmic antigen from molecularly identified cultures [29].

It is clear that these serodiagnostic tests have many limitations, which include the tedious and lengthy preparation of antigens, the fact that the antigens are crude and not standardised, and the cross-reactivity between different mycetoma causative organisms.

### Molecular-based identification methods

Chemical methods, while effective in distinguishing between genera of actinomycetes, are laborious and time consuming. They are complemented and replaced by systematic molecular procedures, notably the 16S rRNA gene sequencing studies [19,30,31]. Other molecular methods championed for this purpose include PCR coupled with restriction endonuclease analyses of PCR products, PCR-based randomly amplified polymorphic DNA fingerprinting, and Curie-point pyrolysis mass spectrometry [32–36]. Such studies allow more accurate classification of strains previously misclassified.

For eumycetoma, various molecular techniques have been used to identify the causative agents, and all are based on the identification of the internal transcribed spacer (ITS). In order to identify all fungal mycetoma causative agents, the ITS regions are usually amplified with panfungal primers and sequenced [34–39]. Identification is based on comparing the resulting sequence with sequences already present in GenBank. Using this approach, several studies reported that a number of causal agents for eumycetoma are underspeciated, as exemplified by the identification of 3 new *Madurella* species (*M*. *fahalii*, *M*. *tropicana*, and *M*. *pseudomycetomatis*) as well as *Pleurostomophoraochracea*, a eumycetoma that produces yellow grains [40,41].

**Fig 12:**
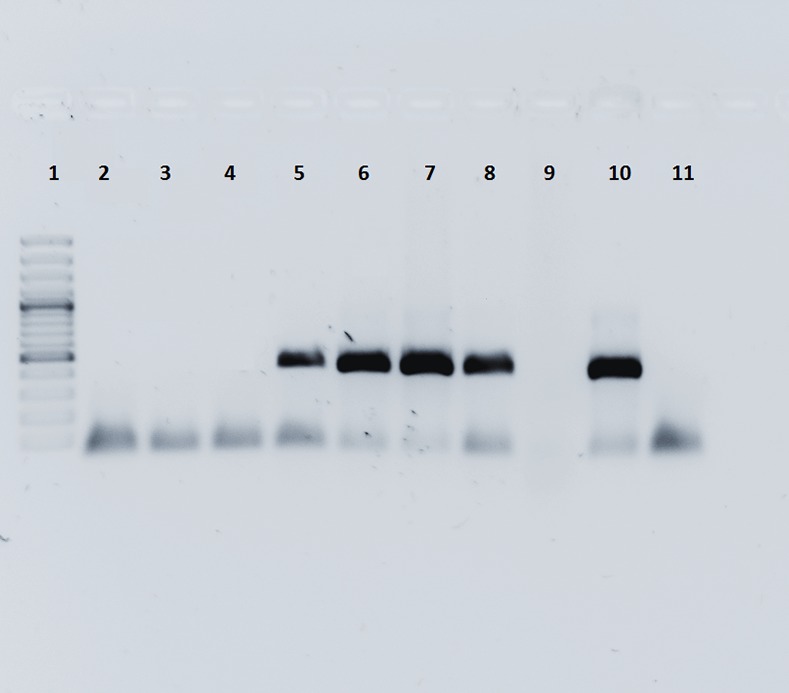
Showing Lane 1 contain a 100 bp DNA ladder. Lanes 2 to 4 show the PCR products for three samples which were negative for Madurella mycetomatis. Lane 5 to 8 shows the PCR products for four samples which were positive for Madurella mycetomatis. Lane 10 was a positive control; lane 11 was a negative control.

For *M*. *mycetomatis*, Ahmed and colleagues developed a species-specific PCR (Fig 12) [34].

This PCR–restriction fragment length polymorphism (RFLP) analysis showed strict homogeneity between *M*. *mycetomatis* isolates, [34] and it can be used to identify the causative agent not only from clinical material but also from environmental samples [42].

Molecular typing of the causative agents can also be performed. For *M*. *mycetomatis*, various methods have been used, including restriction endonuclease analyses (REA), random amplified polymorphic DNA (RAPD), and amplification fragment length polymorphism (AFLP) [34–39]. Although results with RAPD are variable, REA and AFLP were able to differentiate *M*. *mycetomatis* isolates from different countries or even within a country. Certain AFLP types were associated with the origin of the strain or the size of the lesion [34–39].

The genetic variability identification of mycetoma causative organisms is a more stable approach than using methods based on phenotypic criteria. The molecular techniques based on genetic variation are analyses of electrophoretic karyotype differences and RFLPs using gel electrophoresis or DNA–DNA hybridization [34–38].

Prior to DNA extraction, the fungi are usually subcultured on Sabouraud agar and incubated at 37°C for 3 weeks. The mycelia are scraped from the culture medium and homogenised with sterile pestles and mortar. The homogenised mycelia must then be snap frozen in liquid nitrogen, thawed and refrozen twice, and rehomogenised in 2 ml of lysis buffer containing 4 M guanidiniumisothiocyanate, 0.1 M Tris-HCl (pH 6.4), 0.2 M EDTA, and 0.1% Triton X-100. Then DNA should be purified by Celite affinity chromatography [12].

The molecular identification of the causal agents of eumycetoma is mainly based on the ITS that is located between the 18S and 28S genes. The causative agents can be identified to the species level by molecular techniques [34–38].

Using a phylogenetic approach, de Hoog and colleagues studied the natural habitat of *Madurella* species. Four species of *Madurella* were included in a large data set of species of *Chaetomidium*, *Thielavia*, *Chaetomium*, and *Papulaspora* using sequences of the universal fungal barcode gene rDNA ITS and the partial LSU gene sequence. They demonstrated that *Madurella* species are nested within the Chaetomiaceae family [43].

Abdulla and associates in 2003 collected 38 different *M*. *mycetomatis* isolates and analysed the samples by PCR using 20 different primer species. Their result showed a complete lack of DNA fingerprint variation among the various isolates, and they concluded that there is little genetic variation among clinically relevant *M*. *mycetomatis* strains from Sudan [42].

In 2005, van de Sande described the genotyping of *M*. *mycetomatis* by selective AFLP and subtype correlation with the geographical origin and lesion size to discriminate between the strains [39].

Rolling circle amplification (RCA) uses species-specific padlock probes and isothermal DNA amplification, has high specificity and simplicity, and is of low cost. Ahmed and colleagues in 2013 used this technique for the identification of *Falciformispora senegalensis*, *Falciformispora tompkinsii*, *M*. *fahalii*, *M*. *mycetomatis*, *M*. *pseudomycetomatis*, *M*. *tropicana*, *Medicopsis romeroi*, and *T*. *grisea* in a sample of 62 isolates, and the technique produced 100% specificity with no cross-reactivity or false results [37].

Application of isothermal amplification techniques for identification of *M*. *mycetomatis* was used, and the method was found to be reliable and easy to operate. It therefore has the potential to be implemented in areas where mycetoma is endemic. The techniques may be expanded to detect fungal DNA from environmental samples [38].

In general, molecular-based techniques for mycetoma causative organisms are attractive, as they can identify the organism to the species level and thus guide the optimum and appropriate treatment. However, most are expensive, are not field friendly, and are not available in endemic areas [34–39].

Genome sequencing is a new insight into the diagnosis of mycetoma, providing data about the biology and the pathogenicity of the fungi by comparing the genome to other fungi.

*M*. *mycetomatis* mm55 was isolated in 1999 at the Mycetoma Research Centre, Khartoum, Sudan, from an extensive foot mycetoma in a 22-year-old male patient. The strain was isolated by direct culture of the black grains obtained by a deep biopsy and identified by morphology after PCR-RFLP and sequencing of the ITS region. Strain mm55 was sequenced and identified by Smit and colleagues in 2016 [44].

Lucio Vera-Cabrera and associates in 2014 reported on the draft genome sequence of a member of the Thermomonosporaceae family, *A*. *madurae* LIID-AJ290, isolated from a human case of mycetoma. The assembly contains 10,308,866 bps [45].

## Conclusion

In conclusion, accurate identification of the mycetoma causative agent is a prerequisite for the treatment of the disease. Different diagnostic tools are in use to ensure accurate and precise diagnosis of mycetoma. Each method or technique has its advantages and disadvantages, and their specificity and sensitivity are variable. Many factors influence the use of a technique, among which are the type of the lesion, availability of well-equipped laboratory and expert staff, and technique cost. In almost all cases, a combination of techniques and methods is required to reach a correct diagnosis of mycetoma. This is a call for an urgent need for simple, accurate, reliable, cost-effective, and field-friendly diagnostic techniques.

**Fig 13:**
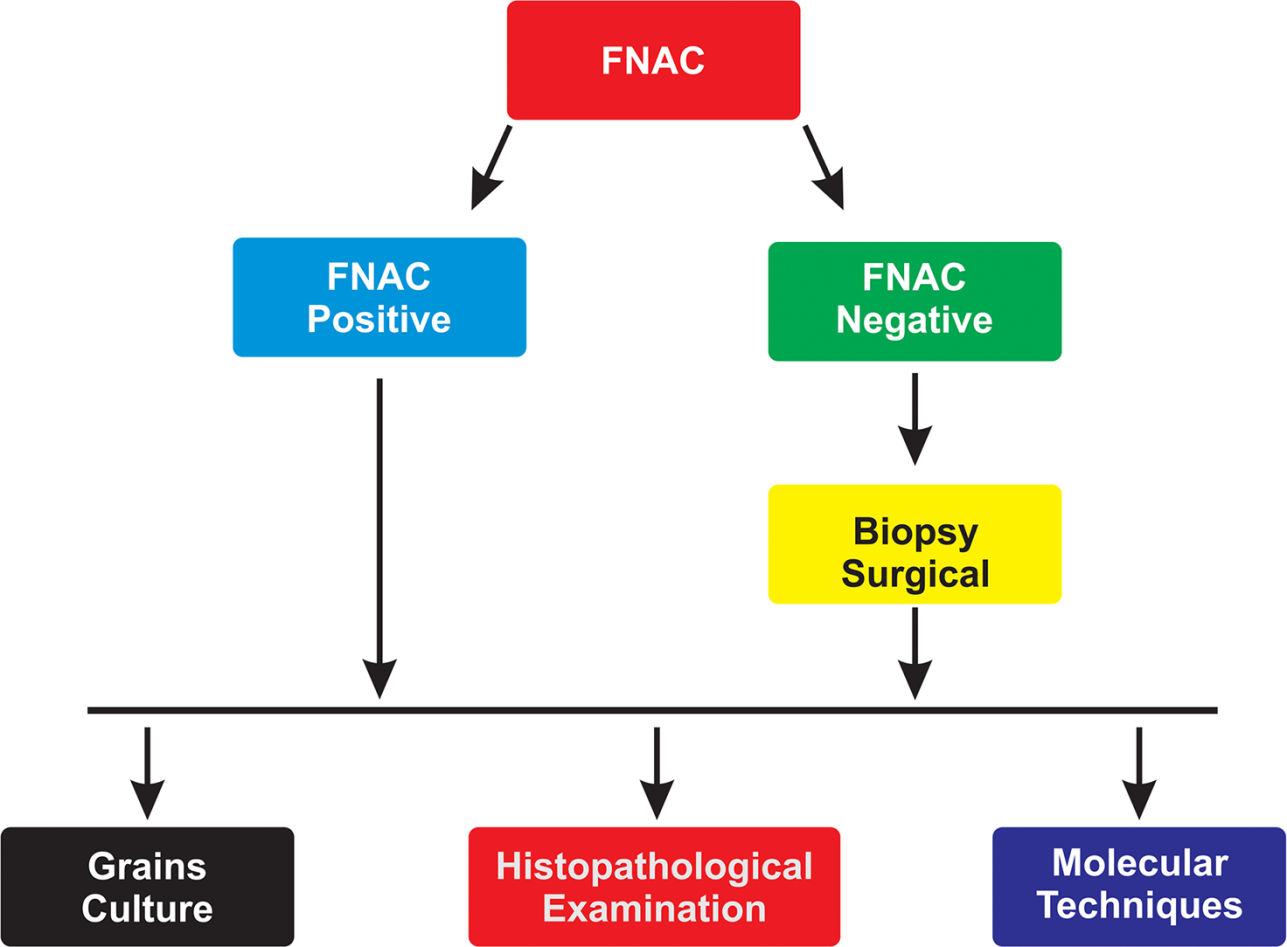
Flow chart for the diagnosis of mycetoma.

In general, the current recommended protocol in endemic areas with meagre resources is to start with FNAC; if the FNAC results are negative, then perform a deep surgical biopsy to obtain grains for culture and conduct a histopathological examination. When available, appropriate molecular techniques molecular identification can be requested (Fig 13).

**Key learning points**

- An ideal diagnostic tool for mycetoma is lacking.
- A combination of techniques is always required to reach a diagnosis.
- The direct microscopy technique is rapid but lacks specificity.
- Cytological examination of a cytological smear is a rapid and simple tool.
- The histopathological technique can give accurate results provided that the grains are available in the tissue section.
- Grain culture in sterile conditions and in expert hands has high yield.
- Molecular techniques in well-equipped centres provide an accurate identification of the causative agents of mycetoma.
- There is an urgent need for simple, accurate, reliable, cost-effective and field-friendly diagnostic tests.

**Top 5 papers**

1. van de Sande WWJ, Fahal AH, Goodfellow M, Mahgoub ES, Welsh O, Zijlstra EE (2014) Merits and Pitfalls of Currently Used Diagnostic Tools in Mycetoma. PLoS Negl Trop Dis 8(7): e2918. doi:10.1371/journal.pntd.0002918
2. van de Sande WWJ, Fahal AH, de Hoog GS, Van Belkum A. (2011) Madurella. In: Liu D, editor. Molecular detection of human fungal pathogens. Boca Raton: CRC Press, Taylor & Francis Group. pp. 117–128.
3. Ahmed SA, van den Ende BH, Fahal AH, van de Sande WW, de Hoog GS. (2014) Rapid identification of black grain eumycetoma causative agents using rolling circle amplification. PLoS Negl Trop Dis. 4;8(12): e3368.
4. Ahmed SA, van de Sande WW, Desnos-Ollivier M, Fahal AH, Mahmoud NA, de Hoog GS. (2015) Application of Isothermal Amplification Techniques for Identification of Madurella mycetomatis, the Prevalent Agent of Human Mycetoma. J Clin Microbiol. 53(10):3280–5.
5. Ahmed AO, Mukhtar MM, Kools-Sijmons M, Fahal AH, de Hoog S, van den Ende BG, et al. (1999) Development of a species-specific PCR RFLP procedure for the identification of Madurella mycetomatis. J Clin Microbiol. 37(10):3175–8.
